# Smurf2 E3 ubiquitin ligase modulates proliferation and invasiveness of breast cancer cells in a CNKSR2 dependent manner

**DOI:** 10.1186/1747-1028-9-2

**Published:** 2014-08-31

**Authors:** Diana David, Sankar Jagadeeshan, Ramkumar Hariharan, Asha Sivakumari Nair, Radhakrishna Madhavan Pillai

**Affiliations:** 1 Research Scholar, Cancer Research, Rajiv Gandhi Centre for Biotechnology, Trivandrum 695 014, Kerala, India; 2 Research Scholar, Department of Genetics, Dr.ALM Post Graduate Institute of Basic Medical Sciences, University of Madras, Chennai, Taramani 600 113, India; 3 Depatment of Pathology, University of Washington, Seattle, WA 98105, USA

**Keywords:** Smurf2, CNKSR2, Oncogenic signaling, PI3K-AKT, Proliferation, Breast cancer

## Abstract

**Background:**

Smurf2 is a member of the HECT family of E3 ubiquitin ligases that play important roles in determining the competence of cells to respond to TGF- β/BMP signaling pathway. However, besides TGF-β/BMP pathway, Smurf2 regulates a repertoire of other signaling pathways ranging from planar cell polarity during embryonic development to cell proliferation, migration, differentiation and senescence. Expression of Smurf2 is found to be dysregulated in many cancers including breast cancer. The purpose of the present study is to examine the effect of Smurf2 knockdown on the tumorigenic potential of human breast cancer cells emphasizing more on proliferative signaling pathway.

**Methods:**

siRNAs targeting different regions of the Smurf2 mRNA were employed to knockdown the expression of Smurf2. The biological effects of synthetic siRNAs on human breast cancer cells were investigated by examining the cell proliferation, migration, invasion, focus formation, anchorage-independent growth, cell cycle arrest, and cell cycle and cell proliferation related protein expressions upon Smurf2 silencing.

**Results:**

Smurf2 silencing in human breast cancer cells resulted in a decreased focus formation potential and clonogenicity as well as *in vitro* cell migration/invasion capabilities. Moreover, knockdown of Smurf2 suppressed cell proliferation. Cell cycle analysis showed that the anti-proliferative effect of Smurf2 siRNA was mediated by arresting cells in the G0/G1 phase, which was caused by decreased expression of cyclin D1and cdk4, followed by upregulation p21 and p27. Furthermore, we demonstrated that silencing of Smurf2 downregulated the proliferation of breast cancer cells by modulating the PI3K- PTEN-AKT-FoxO3a pathway via the scaffold protein CNKSR2 which is involved in RAS-dependent signaling pathways. The present study provides the first evidence that silencing Smurf2 using synthetic siRNAs can regulate the tumorigenic properties of human breast cancer cells in a CNKSR2 dependent manner.

**Conclusions:**

Our results therefore suggest a novel relation between Smurf2 and CNKSR2 thereby regulating AKT-dependent cell proliferation and invasion. Owing to the fact that PI3K-AKT signaling is hyperactivated in various human cancers and that Smurf2 also regulates cellular transformation, our results indicate that Smurf2 may serve as a potential molecule for targeted cancer therapy of certain tumour types including breast cancer.

## Background

The execution of cell division with high fidelity is dependent upon precise spatiotemporal regulation of posttranslational protein modifications. Recently, a flurry of papers reported that E3 ubiquitin ligases perform an integral role in the highly ordered progression of the cell cycle, and that their deregulation contributes to tumorigenesis [1,2]. Smurf2 (Smad ubiquitination regulatory factor 2) is an E3 ubiquitin ligase recently grouped into the Nedd4 family of HECT ubiquitin ligases that negatively regulates TGF-β signaling [3]. In addition to its role in TGF-β signaling, Smurf2 functions in diverse biological pathways, including those controlling the cell cycle and cell polarity/cytoskeletal remodeling. Smurf2 contains WW domains, which directly bind to a PPxY motif (also known as PY motif) in its target. This interaction is further stabilized by the PY tail, a six-amino acid stretch immediately carboxyl terminal to the PPxY motif, although additional interactions exist [4]. For further insight into the cell cycle-regulatory role of Smurf2, we used a homology-based approach to select for potential Smurf2 interactors, examining those proteins that contain a PPxY-motif.

Nonetheless, it was reported that Smurf2 perform a dual role in cancer by functioning as both tumor promoter and suppressor by controlling the stability of several important proteins with central role in cell-cycle progression, proliferation, differentiation, metastasis, genomic stability and senescence. Notably, aberrant expression of Smurf2 occurs in several types of cancers, including breast, esophageal, pancreatic and renal cell carcinomas. In contrast, Smurf2 was found to induce senescence and recent mouse model studies by Blank et al. [5] showed that Smurf2 is a bona fide tumor suppressor, as the Smurf2-deficient mice are prone to a variety of cancers, including lymphoma, hepatocellular, lung and mammary carcinoma. Since Smurf2 is considered to play a contradictory role as tumor promoter and suppressor, understanding the biological functions of Smurf2 and its associated regulatory networks would be crucial for providing insights into the mechanisms of Smurf-mediated cancer progression and also in developing therapeutic strategies that target the Smurf pathway in human cancers [6].

Through their role as a regulator of TGF-β mediated transcriptional events, Smurfs have been tangentially implicated in the control of the cell cycle. Interestingly, it was observed that unlike Smurf1 which is expressed constantly throughout the cell cycle, expression and localization of Smurf2 itself is cell cycle regulated which accumulates during late G2 through early mitosis and is mainly localized to the centrosome from G1 through prophase, then localizes to the spindle midzone during anaphase and the midbody during cytokinesis. This localization pattern of Smurf2 implicates a predominant role for Smurf2 in regulating cell cycle progression. Furthermore, acute depletion of Smurf2 in mammalian cells leads to multinucleation and often initiates chromosomal misalignment at metaphase and premature onset of anaphase with defective chromosome segregation and cytokinesis [1]. It has been shown that Smurfs regulate the expression of various mammalian proteins that control cell-cycle progression, including Mad2 [1] NEDD9-Aurora A [4,7], RhoA [7], KLF2 [8] and KLF5 [9,10]. All these data strongly support the role of Smurf2 in tumorigenesis, and subsequently blocking Smurf2 expression would be a rational strategy to treat breast cancer.

Among various strategies employed to inhibit gene expression, RNA interference (RNAi) offers significant promise for cancer therapy due to its ability to potently knockdown a specific gene. siRNAs of 20 to 25 nucleotides in length silences a target gene by binding to its complementary mRNA and triggering its degradation [11-13]. In the present study, we have evaluated the effect of Smurf2 silencing on colonigenicity, invasive properties, proliferation, and cell cycle in breast cancer cells using synthetic siRNA.

## Results

### Expression of Smurf2 is dysregulated in human breast cancer tissues and cell lines

**Figure 1:**
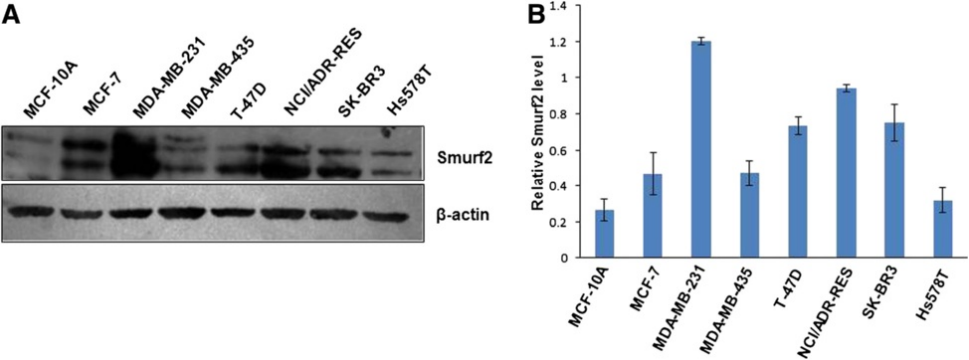
**Smurf2 is upregulated in human breast cancer cell lines. (A)** Smurf2 was found to be specifically upregulated in MDA-MB-231 cell line compared to other breast cancer cell lines. An untransformed immortalized cell line, MCF-10A was used as the control. β-actin was used to verify equal gel loading. **(B)** The bar graph indicates relative levels for Smurf2 protein in cancer cell lines to that in MCF10A. The density of each Smurf2 signal was normalized by β-actin. Data shows mean value ± S.E. from three independent experiments.

In order to explore the role of Smurf2 in carcinogenesis, we first screened for Smurf2 expression in different cancer cell lines by western blot and observed an elevated expression of Smurf2 in MDA-MB-231 breast cancer cell line compared to others [6]. Hence, to determine the appropriate model system for our *in vitro* study, we delineated the expression of Smurf2 protein in seven breast cancer cell lines. As control, we included an untransformed but immortalized MCF-10A cell line in the study. As reported previously [14], we also observed that Smurf2 expression was decreased in MCF10A cells however, a strong up-regulation was observed in MDA-MB-231 cells compared to other cancer cell lines (Figure 1). Similarly, tissue level expression of Smurf2 was also analyzed by western blot and it was observed that human breast IDCs (Infiltrating ductal carcinoma) showed elevated constitutive expression of Smurf2 when compared to normal counterparts [6]. Together, these results suggested that elevated Smurf2 levels in breast tumours and cancer cell lines might contribute to the transforming property of human breast cells.

### Silencing of Smurf2 gene by predesigned siRNAs

**Figure 2:**
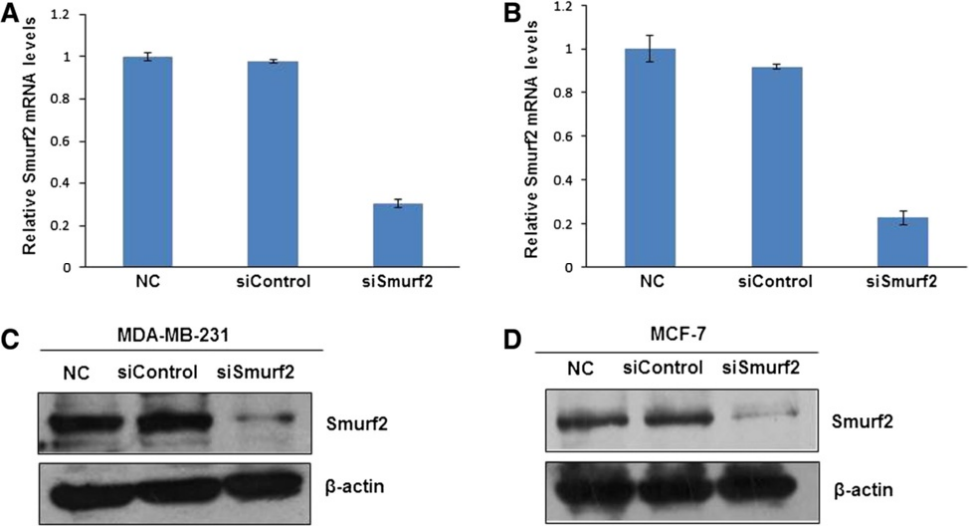
**Knockdown effect of Smurf2 siRNA in MDA-MB-231 and MCF-7 cells. (A)** MDA-MB-231 cells were transfected with Smurf2 siRNA (siSmurf2) and control siRNA (siControl) at a concentration of 80 pmols. Cells were harvested 36 hours after the transfection, and the silencing effect at the Smurf2 mRNA level was determined using real-time RT-PCR. **(B)** MCF-7 cells were transfected with Smurf2 siRNA, and control siRNA. The silencing effect at the Smurf2 mRNA level was measured using real-time RT-PCR. The silencing effect of Smurf2 siRNA at the protein level was determined 48 hours post-transfection using western blot in **(C)** MDA-MB-231 cells and **(D)** MCF-7 cells.

To silence Smurf2 expression, a mixture of three target specific 20–25 nt siRNAs targeting different regions of Smurf2 or the negative control siRNA containing a scambled sequence which will not lead to the specific degradation of any known cellular mRNA included in the kit were transfected to MDA-MB-231 cells at a concentration of 80 pmols with siLentFect reagent. Smurf2 siRNA showed a significant silencing effect and knocked down 78% of Smurf2 mRNA in comparison with control siRNA (Figure 2A). Considering the fact that siRNA transfection efficiency may vary in different cell lines, we also examined the silencing effect of Smurf2 siRNA in MCF-7 cells. Approximately 69% of Smurf2 mRNA were silenced in MCF-7 cells after treatment with Smurf2 siRNA (Figure 2B), respectively. The silencing effect of Smurf2 expression at the protein level was also confirmed with western blot. Smurf2 siRNA significantly inhibited the Smurf2 protein expression in MDA-MB-231 cells and MCF-7 cells, which is consistent with the silencing effect at the mRNA level (Figure 2C, D).

### Smurf2 silencing inhibits focus formation of breast cancer cells

**Figure 3:**
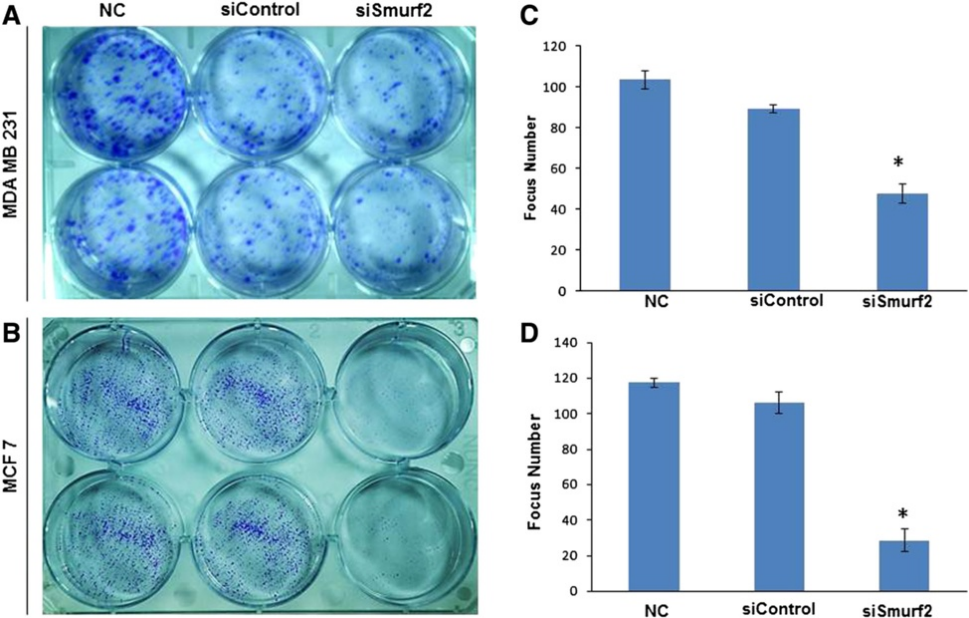
**Silencing of Smurf2 inhibits focus formation.** Forty-eight hours after the siRNA transfection, **(A)** MDA-MB-231 cells and **(B)** MCF-7 cells were seeded in six-well plates, and the medium was changed every 2 days. Cells cultured for 14 days were washed twice with 1xPBS, fixed by 4% paraformaldehyde, and stained with 0.5% crystal violet. Images of the colonies were obtained with a digital camera. **(C)**, **(D)** Foci containing ≥50 cells were counted for MDA-MB-231 and MCF-7 cells and the result represented as mean ± standard deviation (n = 3). *P < 0.05 compared with control siRNA. NC-Negative control without siRNA.

First, we used a focus formation assay to test whether silencing Smurf2 in breast cancer cells affects the clonogenic potential, which correlates with tumor formation *in vivo*[15]. Forty-eight hours after transfection, a single-cell suspension was seeded into six-well plates and incubated for 14 days to allow focus formation. The cells were fixed, stained with crystal violet, and counted. Foci containing ≥ 50 cells were counted manually. As Figure 3A shows, MDA-MB-231cells treated with Smurf2 siRNA exhibited smaller focus diameter as well as focus numbers compared with cells treated with the control siRNA. However, in MCF-7 cells the effect of siRNA is drastically increased, causing a considerable decrease both in focus number and size compared with MDA-MB-231 cells treated with Smurf2 siRNA (Figure 3B). These data indicated that inhibition of Smurf2 significantly decreases the cells’ focus formation potential, which correlates with the formation of tumors in nude mice [16].

### Anchorage-independent growth of breast cancer cells hindered by Smurf2 silencing

**Figure 4:**
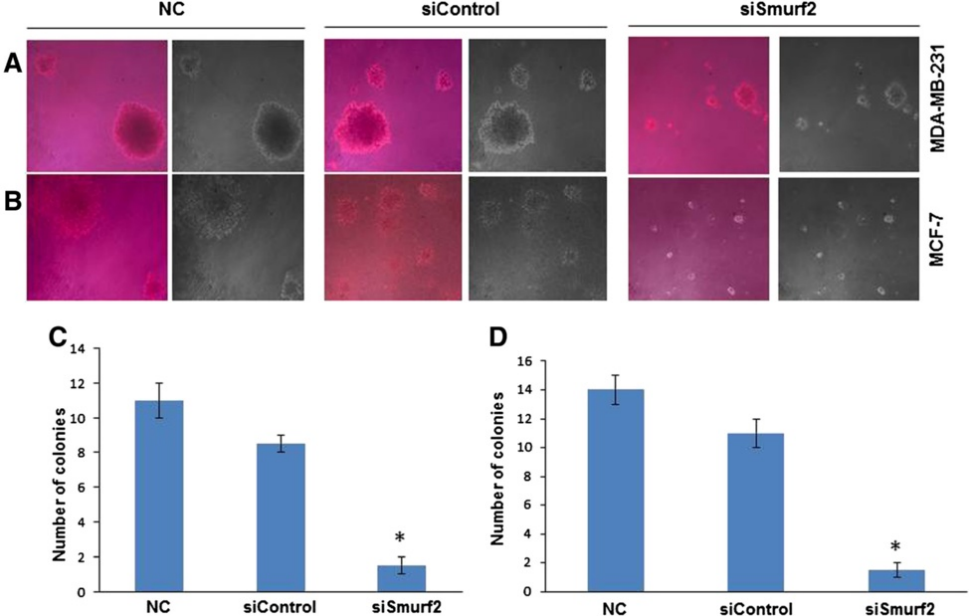
**Smurf2 silencing inhibits colony formation.** A soft agar assay was conducted to examine the colony formation ability of **(A)** MDA-MB-231 cells and **(B)** MCF-7 cells. Twenty-four hours after the siRNA transfection, MDA-MB-231 and MCF-7 cells were seeded in 0.35% agarose in DMEM medium supplemented with 10% FBS at a density of 1 × 10^3^ per 35-mm culture dish and allowed to grow for 21 days. The dishes were stained with 0.01% crystal violet, and the colonies were examined with microscope. Results are representative picture of colonies of two independent experiments done in triplicate. **(C)**, **(D)** The number of colonies of MDA-MB-231 and MCF-7 cells were counted and the result represented as mean ± standard deviation (n = 3). *P < 0.05 compared with control siRNA. NC-Negative control without siRNA.

Anchorage-independent growth in the semisolid medium of soft agar is a strong indicator of a transformed phenotype [16]. In order to examine whether Smurf2 knockdown can influence the anchorage-independent growth potential, we performed a soft agar assay in MDA-MB-231 and MCF-7 cells. Twenty four hours after the transfection, a single-cell suspension was seeded into 0.35% agarose supplemented with DMEM medium and 10% FBS. The cells were cultured for another 21 days under normal cell culture conditions to allow colony formation. As shown in Figures 4A and C, silencing Smurf2 in MDA-MB-231 cells dramatically inhibited the transformed phenotype. Individual colony size was much smaller in Smurf2 siRNA transfected cells compared with control siRNA-treated cells. Similar results were also observed in MCF7 cells (Figures 4B and D). This result indicated that silencing of Smurf2 in breast cancer cells suppress anchorage-independent growth capability.

### Smurf2 silencing impedes cell motility and invasion

**Figure 5:**
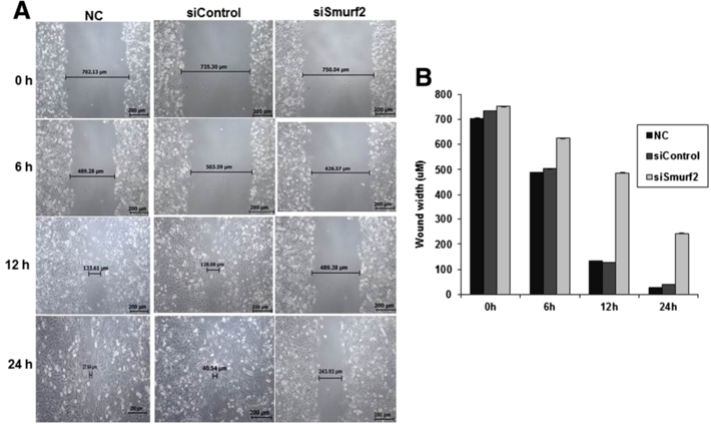
**Smurf2 knockdown impairs cell motility. (A)** Wound healing assay was done to evaluate the migration potential of MDA-MB-231 cells after silencing Smurf2 expression. Fifty-six hours after the transfection of siRNA, cells were wounded and monitored with a microscope every 6 hours. **(B)** The migration was determined by the rate of cells filling the scratched area and the result represented as mean ± standard deviation (n = 3). NC-Negative control without siRNA.

**Figure 6:**
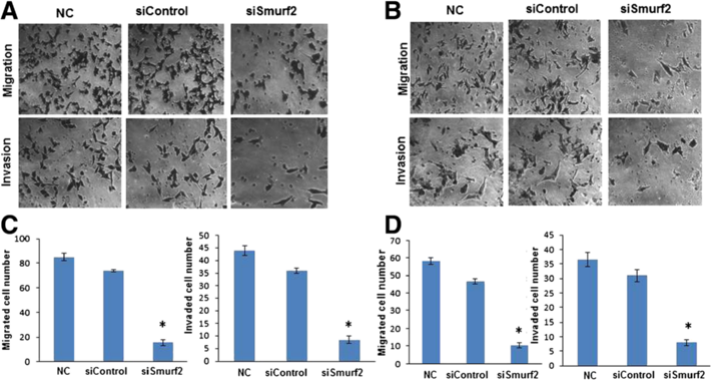
**Silencing of Smurf2 expression inhibits migration and invasion abilities of MDA-MB-231 and MCF-7 cells.** Cell migration was determined using Boyden transwell chambers. Forty-eight hours after the transfection with siRNA, **(A)** MDA-MB-231 cells and **(B)** MCF-7 cells were suspended in serum-free medium and seeded on 24-well transwell plates. Cells migrated though pores to the bottom surface of the transwell were fixed with with 10% formaldehyde, and stained with 0.5% crystal violet and counted. Six random microscopic fields were counted for each group. Cell invasion was assayed in transwell coated with Matrigel. Invaded cells were fixed, stained and counted. Six random microscopic fields were counted for each group. All experiments were performed in triplicates and repeated three times. Significant reduction of migration and invasion was observed after silencing Smurf2 expression in MDA-MB-231 and MCF-7 cells. **(C), (D)** The number of migrated or invaded cells of MDA-MB-231 and MCF-7 were counted from five or six randomly selected fields in a blind way and the result represented as mean ± standard deviation (n = 3). *P < 0.05 compared with control siRNA. NC, negative control without siRNA.

Reduced clonogenic potential is usually associated with the loss of invasion capabilities in tumor cells [15,16]. Therefore, we analyzed the cell motility of breast cancer cells using a classic wound healing assay in which the cell monolayer was scratched and cells migrating to the wound area were monitored at different time points. Compared with cells transfected with control siRNA, the cells treated with Smurf2 siRNA showed a wider wound area 24 hours after wound generation, and took a longer time to fill in the wound area, indicating a defect in migration (Figure 5). Since both cell migration and invasion have decisive role in the dissemination of cancer cells and metastases, we further investigated the cell invasiveness using *in vitro* migration and invasion assays. Migration assay was done as described previously [14] using uncoated Boyden chamber to examine the *in vitro* migration ability of tumor cells. Cells that migrated to the bottom of the transwell were fixed, stained and counted. Compared with the control group, Smurf2 siRNA transfected cells showed a significant decrease in the number of migrated cells in MDA-MB-231 (Figure 6A, C) and MCF-7 cells (Figure 6B, D). Additionally, matrigel coated transwell chambers were used to access the invasive potential of breast cancer cells. Consistent with the finding in migration assay, MDA-MB-231 (Figure 6A, C) and MCF-7 (Figure 6B, D) cells treated with Smurf2 siRNA exhibited a significant reduction in cell invasion ability in comparison with control siRNA-treated cells. Collectively, these results imply that silencing of Smurf2 decreases the invasive properties of breast cancer cells.

### Downregulation of the proliferative potential in breast cancer cells post Smurf2 silencing

**Figure 7:**
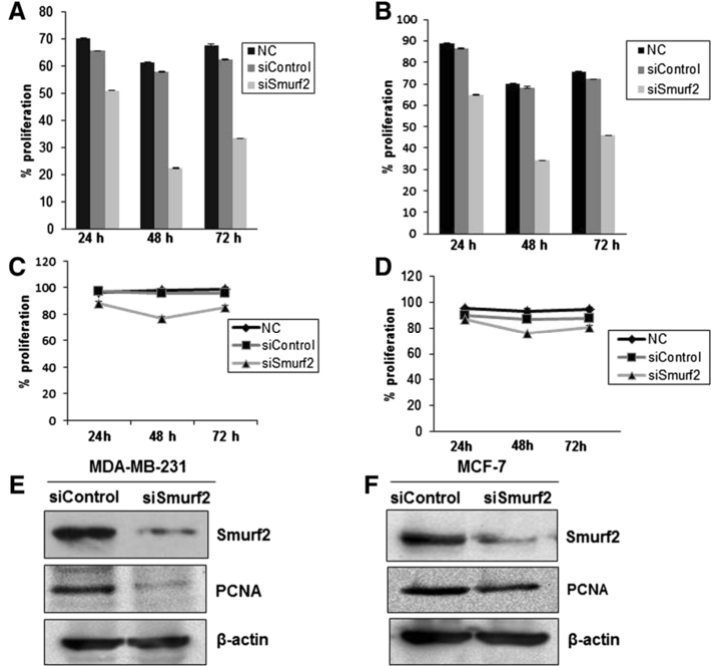
**Smurf2 knockdown downregulates cell proliferation.** Smurf2 knockdown decreases proliferation of MDA-MB-231 and MCF-7 cells which was measured with an MTT [3-(4,5-dimethylthiazol-2-yl)-2,5-diphenyl tetrazolium bromide] assay **(A, B)** and BrdU (5-bromo-2′-deoxyuridine) cell proliferation assay **(C, D)**. Result represented as mean ± standard deviation (n = 3). The inhibition effect on cell proliferation is more significant at 48 hours rather than 72 hours posttransfection, after which proliferation gradually increased, probably due to transient transfection. Expression of PCNA (Proliferating cell nuclear antigen) was found to be downregulated in Smurf2 knockdown cells of **(E)** MDA-MB-231 and **(F)** MCF-7, 48 hours posttransfection.

**Figure 8:**
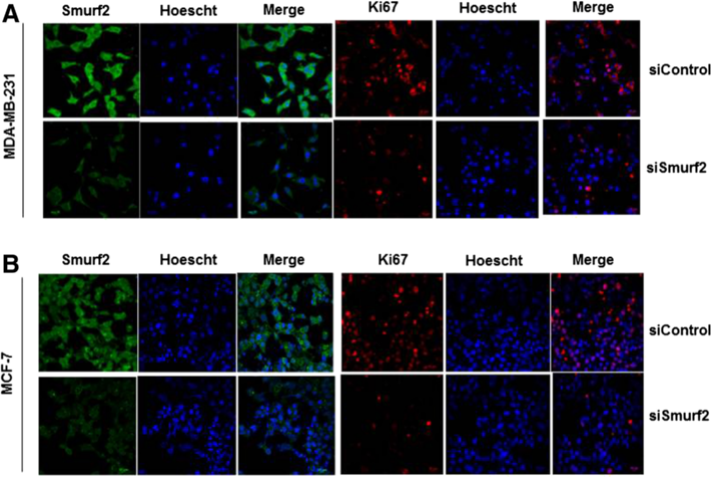
**Silencing of Smurf2 downregulates Ki-67 expression.** Smurf2 knockdown significantly downregulated the expression of the proliferation marker Ki-67 in **(A)** MDA-MB-231 and **(B)** MCF-7 cells compared to control siRNA treated cells.

Carcinogenesis is a multistage process initiated by disturbed and uncontrolled proliferation of cells [17]. In order to address whether Smurf2 is essential for the proliferation of breast cancer epithelial cells, we next examined the proliferation rate of breast cancer cells after silencing of Smurf2 with siRNA. Cell growth was determined at 24, 48 and 72 hours post-transfection. Compared with cells transfected with the control siRNA, cells treated with Smurf2 siRNAs demonstrated lower viability and slower growth rate (Figure 7A, B, C, D). Moreover, the inhibition effect on cell proliferation is more significant at 48 hours rather than 72 hours posttransfection. Eventhough the proliferation rate increases slightly after 72 hours post-transfection it is significantly lower compared to 24 hours post-transfection. Furthermore, the inhibitory effect of Smurf2 on breast cancer cell proliferation was confirmed by using proliferation markers such as PCNA and Ki67 which are important regulators of proliferative indices [18]. The expression of PCNA, which increases during the G1-phase, peaks at the S-phase and declines during G2/M-phase of the cell cycle was found to be downregulated in Smurf2 siRNA treated cells compared with scrambled siRNA treated cells (Figure 7E, F). Consistently, the expression of another specific proliferation marker, antigen Ki-67 which is a ubiquitous human nuclear protein expressed in G1, S, and G2 phases of the cell cycle but not in the G0-phase was also found to be significantly downregulated in Smurf2 siRNA treated cells compared to control siRNA treated cells (Figure 8A, B). These results suggested the pivotal role of Smurf2 in the proliferation and survival of breast cancer cells, and that suppression of Smurf2 could lead to downregulation of cell proliferation.

### Smurf2 knockdown induces cell arrest in G0/G1 phase

**Figure 9:**
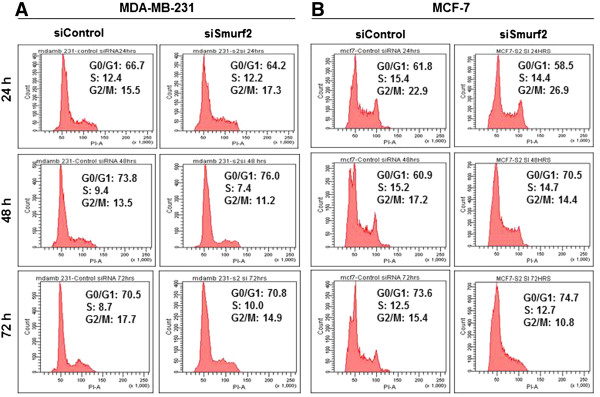
**Silencing of Smurf2 leads to G0/G1 phase arrest.** Cell cycle distribution of **(A)** MDA-MB-231 cells and **(B)** MCF-7 cells treated with Smurf2 siRNA and control siRNA were accessed by flow cytometry at 24, 48, and 72 hours post-transfection. Results are representative histogram of three independent experiments, plotting cell count vs. DNA content.

To identify the mechanism for this anti-proliferation effect, we investigated the cell cycle distribution of breast cancer cells after silencing Smurf2 expression in MCF-7 and MDA-MB-231. As shown in Figure 9, cells transfected with Smurf2 siRNA induced a significant G_0_/G_1_ block in comparison with cells treated with control siRNA, specifically 48 hours post-transfection. The effect of Smurf2 depletion on cell growth was not due to increased cell death, as a sub-G_0_ peak was not detected in Smurf2 siRNA cells. In addition, we did not observe any profound S or G_2_-M cell cycle block in Smurf2-silenced cells. However, a slightly increased number of Smurf2-depleted MDA-MB-231 cells were consistently identified in G_0_/G_1_ at 24, 48 and 72 hours (64.2%, 76%, and 70.8%), in comparison with cells treated with control siRNA (66.7%, 73.8%, and 70.5%) (Figure 9A), which suggested that Smurf2 silencing may have caused cells to accumulate in G_0_/G_1_. In the same experiment, a comparatively similar result was observed in MCF-7 cells (Figure 9B). The percentages of cells in the G_0_/G_1_ phase were 58.5%, 70.5%, and 74.7% for cells treated with Smurf2 siRNA at 24, 48 and 72 hours respectively. In comparison, only 61.8%, 60.9%, and 73.6% of MCF-7 cells treated with scrambled siRNA at 24, 48 and 72 hours were in the G_0_/G_1_ phase. These findings indicated that Smurf2 depletion had a significant cytostatic effect on breast cancer cell lines, as Smurf2 siRNA markedly inhibited cell proliferation via blocking cell cycle progression at the G_0_/G_1_ phase but had a negligible effect on cell death.

**Figure 10:**
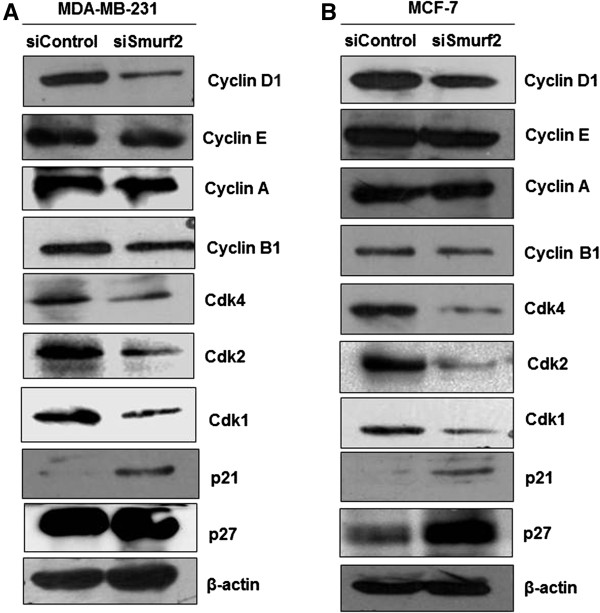
**Expression levels of cell cycle regulatory proteins following Smurf2 knockdown.** Silencing Smurf2 expression induces accumulation of cells in the G_0_/G_1_ phase. Lysates from **(A)** MDA-MB-231 and **(B)** MCF-7 cells following Smurf2 siRNA treatment were probed with the indicated antibodies. β-actin was used as the loading control.

Cell cycle progression is driven by the oscillating activation of CDKs alongside precisely timed fluctuations in the synthesis and degradation of cyclins [19]. Since cyclin D1 was reported to play a significant regulatory role during progression through the G_1_ phase of breast cancer cells [20], we next examined whether the expression of cyclin D1 was responsible for the G_0_/G_1_ cell cycle arrest in Smurf2 siRNA-treated cells. As Figure 10A, B indicates, silencing of Smurf2 significantly decreased the expression of cyclin D1 in breast cancer cells. However, the expression of cyclin A, cyclin E and cyclin B1 remain unchanged. The D-type cyclins are able to bind to several different CDK partners; Cdk2, Cdk4, Cdk5 and Cdk6. Of these, their main and consistent partner appears to be Cdk4 which associates in late G_1_ and early S phase [20]. Interestingly, we observed that, in Smurf2 siRNA treated cells, the levels of Cdk4 were found to be significantly downregulated, in comparison to control siRNA treated cells. A concomitant decrease was also observed in the expression levels of Cdk2 and Cdk1 following Smurf2 knock down in MCF7 and MDA-MB-231 cells. However, the expression of p27 and p21 cyclin dependent kinase inhibitors (CKIs) were found to be constitutively elevated following silencing of Smurf2 in MCF7 and MDA-MB-231 cells. It is widely accepted that elevated levels of p21cip1 and p27kip1 induce G_1_ arrest; therefore, it is highly likely that the impaired G_1_-S transition noted in Smurf2-depleted cells occurs as a result of deregulation of p21^cip1^ and p27^kip1^ levels [17,20]. All together, these findings suggests that Smurf2 silencing modulates signaling pathways that are integral to G_1_-S progression, resulting in constitutively high levels of p21^cip1^ and p27^kip1^ that block cell cycle progression.

### Depletion of Smurf2 destabilizes CNKSR2

**Figure 11:**
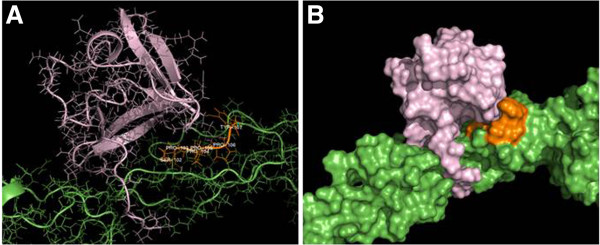
**Smurf2-WW2 domain interacts with CNKSR2. (A)**, **(B)** Docking of Smurf2-WW2/3 domains (purple coloured) and CNKSR2 (green coloured) using PATHDOCK and GROMACS indicate that the Smurf2-WW2 domain shows a better penetration and more stabilization with ‘SPPPPY’ motif (orange coloured) at 702–707 sequence region of CNKSR2, than Smurf2-WW3 domain.

**Figure 12:**
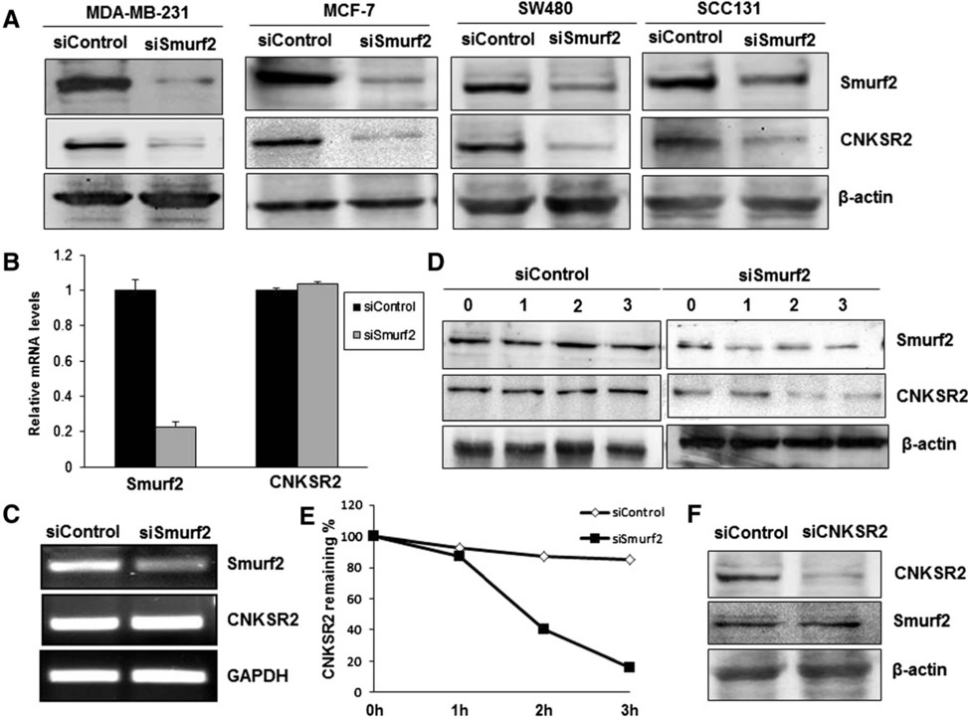
**Depletion of Smurf2 accelerates degradation of CNKSR2. (A)** Depletion of Smurf2 by siRNA (siSmurf2) leads to decreased CNKSR2 protein levels in MDA-MB-231, MCF-7, SW480, and SCC131 cells. **(B)** Smurf2 does not affect CNKSR2 transcript level. RNA samples from MDA-MB-231 cells transfected with siSmurf2 were analyzed by qRT-PCR and further by **(C)** RT-PCR for the indicated transcripts. **(D)** MDA-MB-231 cells transfected with control siRNA and Smurf2 siRNA were treated with cycloheximide at 100 μg/ml, 48 hours post-transfection. Cells were harvested 0, 1, 2, or 3 h after cycloheximide addition and the cell lysate was subjected to SDS-PAGE followed by immunoblotting with specific antibodies. **(E)** Plot of CNKSR2 degradation rate was shown in the panel. **(F)** CNKSR2 depletion does not affect Smurf2 levels.

Cell proliferation is regulated by multiple pathways such as the Raf-MEK-ERK, NF-kB or phosphatidylinositol- 3 kinase (PI3K)-AKT pathways [21]. In order to identify novel interacting partners of Smurf2 involved in cell proliferation we used a homology-based approach to select for potential Smurf2 interactors, examining those proteins that contain a PPxY-motif. Strikingly, we identified CNKSR2 (Connector enhancer of kinase suppressor of ras2) which possess a ‘SPPPPY’ motif at 702–707 sequence region that shows a strong PY motif match with Smurf2 [see Additional file 1: Table S1]. Further studies using PATHDOCK and GROMACS provided an insight into the interaction between CNKSR2 and WW domains of Smurf2. It was observed that WW2 domain of Smurf2 can penetrate more and stabilize with ‘SPPPPY’ motif of CNSRK2 compared with Smurf2-WW3 domain (Figure 11, see Additional file 2: Table S2). The possible interaction between Smurf2 and CNKSR2 has to be further evaluated in detail. CNK (Connector enhancer of ksr) proteins are evolutionarily conserved scaffold proteins essential for different signaling pathways. CNKSR2, the human homolog most resembling Drosophila CNK, modulates the Raf–MEK–ERK pathway in neuronal cells and is involved in neuronal cell proliferation and differentiation. It regulates the RAS-dependent signaling pathways upstream or in parallel to RAF, especially in RAF compartmentalization and operates in several RTK-mediated developmental events affecting cell proliferation/survival, differentiation and migration [22].In our study we observed that Smurf2 knockdown modulates the level of CNKSR2. In particular, we addressed whether the E3 ligase Smurf2 would directly target CNKSR2 for proteasome-mediated degradation. Contrary to this speculation, it was consistently observed that CNKSR2 protein levels were decreased by siRNA mediated Smurf2 depletion in MDA-MB-231 and MCF7 breast cancer cell lines, SW480 colon cancer cell line and SCC131 oral cancer cell line (Figure 12A). The decline in CNKSR2 protein levels induced by Smurf2 depletion was a post-transcriptional effect as qRT-PCR analysis showed little to no effect on CNKSR2 mRNA level (Figure 12B, C), suggesting Smurf2 controls the CNKSR2 protein level possibly through proteolytic regulation. To confirm a role for Smurf2 in regulating CNKSR2 degradation, we employed a cycloheximide- based protein degradation assay. After protein synthesis was blocked by cycloheximide, CNKSR2 degraded more rapidly in the Smurf2 depleted cells (Figure 12D, E). These results show that Smurf2 plays an essential role in maintaining the stability of CNKSR2 protein. To reciprocally establish whether CNKSR2 controls Smurf2 levels, MDA-MB-231 cells were transfected with CNKSR2 siRNA. CNKSR2 depletion had no discernable effect on Smurf2 protein levels (Figure 12F). These data suggest that Smurf2 positively regulates the level of CNKSR2 protein at the post-transcriptional level.

### Smurf2 regulates cell proliferation through a CNKSR2-AKT-FoxO3a dependent pathway

**Figure 13:**
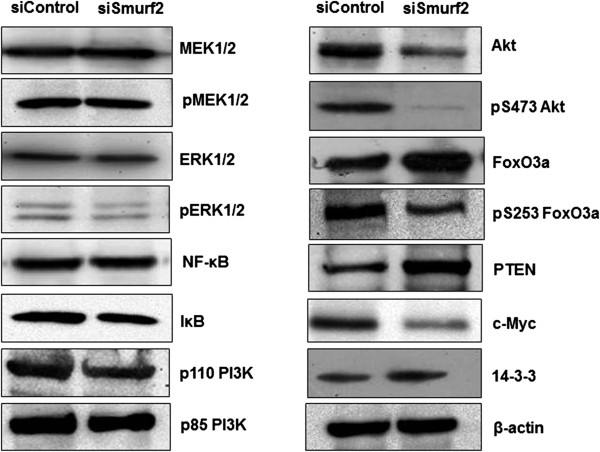
**Smurf2 knockdown modulates the PI3K-AKT signaling pathway in a CNKSR2 dependent manner.** Smurf2 knockdown diminishes Akt phosphorylation and FoxO3a-dependent cell proliferation. Lysates from control siRNA and Smurf2 siRNA treated MDA-MB-231 cells were probed with the indicated antibodies. β-actin was used as the loading control.

The PI3K pathway, activated by receptor tyrosine kinase growth factors provides proliferative and antiapoptotic signals and is frequently deregulated and/or activated in human cancers. The PI3K activity is also required for G1/S phase progression in lymphocytes and in human mammary epithelial cells (HMECs) [23-25]. AKT is an important downstream effector of PI3K. AKT regulates proliferation as well as cell survival and PI3K-AKT signaling is frequently hyperactivated in human tumours including breast cancer [21]. Since CNKSR2 plays an upstream regulatory role in RAS-mediated signaling pathways [22,26,27], we analyzed the expression of MEK1/2, pMEK1/2, ERK1/2, pERK1/2 and NF-kB after Smurf2 knockdown. However, downregulation of CNKSR2 does not interfere with Ras-MEK-ERK and NF-kB signaling in MDA-MB-231 cells, which overexpress a constitutively active Ras molecule. To check whether CNKSR2 influences AKT activity, we analyzed the phosphorylation status of AKT at S473 in MDA-MB-231 cells. This phosphorylation is crucial for AKT activity [28]. We observed reduced levels of phosphorylated AKT in Smurf2-knockdown cells (Figure 13). In addition, the expression of PI3K catalytic subunit p110 was found to be slightly downregulated in Smurf2 knockdown cells, however there is not much variation in the expression levels of the regulatory subunit p85 which is required for the stabilization and localization of p110-PI3K activity [29]. AKT fosters proliferation through phosphorylation of various anti-proliferative regulators such as FoxO transcription factors. Results from previous studies have shown that AKT mediated phosphorylation of FoxO3a is critical for its DNA-binding and transcriptional activity. Specifically, FoxO3a activity is negatively regulated by AKT, which phosphorylates FoxO3a at multiple sites, facilitating its association with 14-3-3 protein, thereby leading to its transport out of the nucleus and retention in the cytoplasm thereby preventing FoxO-dependent transcriptional activation and thus promoting cell proliferation [21,30]. Interestingly, we observed that Smurf2 knockdown decreased FoxO3a phosphorylation at S253. However, the expression of total FoxO3a was found to be slightly upregulated following Smurf2 knockdown. Impaired Akt activity gauged by the marked decrease in p-FoxO3a(S253) expression may contribute to the retention of FoxO3a in the nucleus of Smurf2 knockdown cells, thereby promoting its tumor suppressor functions, by upregulating the expression of FoxO-responsive genes such as p27/Kip1 and p21/waf1. All these data support the physiological relevance of CNKSR2 in AKT-dependent regulation of FoxO3a activity in MDA-MB-231 cells. No significant effect was observed on the protein levels of total 14-3-3.

Interestingly, we also observed that expression of a potent oncogene, c-Myc was found to be downregulated in Smurf2 knockdown cells (Figure 13). The c-myc gene is amplified in various human cancers, acts as a transcriptional regulator, and is overexpressed in many types of human cancers and is also reported to indirectly inhibit PTEN expression [31]. Downregulation of c-Myc was followed by a concomitant increase in the expression of PTEN (Figure 13) which might be responsible for the decreased phosphorylation of AKT at S473 which is consistent with the well-established inverse relationship between MMAC/PTEN expression and AKT activation [32]. Thus Smurf2 knockdown probably downregulates proliferation of breast cancer cells in a CNKSR2 dependent manner by modulating the PI3K- PTEN-AKT-FoxO3a pathway.

## Discussion

Smurf2 plays a decisive role in TGF-β/BMP signaling, cell migration, cell polarity, differentiation and senescence, mainly by targeting corresponding cellular substrates for ubiquitination and proteasomal degradation. Smurf2 has been found to be upregulated in several types of cancer including breast cancer and has been associated with poor prognosis in esophageal squamous cell carcinoma and renal cell carcinoma [6]. Our findings support the hypothesis that Smurf2 plays a conspicuous role in the tumorigenesis of breast cancer.

Initially, Smurf2 has been found to play an important role in cellular transformation by regulating the TGF-β/BMP signaling, deregulation of which will invariably lead to developmental defects and/or diseases, including cancer [33]. In addition, Smurf2 plays a pivotal role in proliferating cells by controlling various protein complexes, critical for cell division and growth, such as KLF2, KLF5, NEDD9-Aurora A etc. [6]. Hence we were interested to determine the effect of Smurf2 knockdown on proliferation of breast cancer cells by analysing the focus formation and colony formation ability in soft agar compared with cells transfected with the control siRNA. Interestingly, we observed that silencing of Smurf2 with siRNA led to significant reduction in focus formation and colony formation in both MCF-7 cells and MDA-MB-231 cells (Figures 3 and 4).

Several lines of evidence implicate that Smurf2 and its interacting partners or substrates are involved in cell invasion and tumour metastasis [6]. Recently, Jin et al. reported that upregulation of Smurf2 promotes metastasis of breast cancer cells by enhancing migration and invasiveness specifically by up-regulating the expression of N-cadherin which is involved in epithelial-mesenchymal transition, in a TGF-β/Smad independent manner [14]. Concomitantly, we performed numerous experiments including the wound healing assay, migration assay, and invasion assay to assess the effect of Smurf2 knockdown on invasive potential of MCF7 and MDA-MB-231 breast cancer cells. As shown in Figures 5 and 6, the invasiveness properties were significantly inhibited in cells treated with the Smurf2 siRNA in comparison with cells treated with the control siRNA. These data are consistent with the previous report that inhibition of Smurf2 expression in breast cancer cells induced a less invasive phenotype compared with cells transfected with control siRNA.

Smurf2 possess WW domains, which mediate interactions with proteins that have PPxY motifs [34]. In order to identify novel interacting partners of Smurf2 which may have crucial role in cell proliferation, we did a homology-based approach and surprisingly we identified a scaffold protein CNKSR2 involved in Ras-Raf mediated signaling pathways. Although the relationship between Smurf2 and CNKSR2 has not been identified yet, the presence of a PPxY motif in its structure (SPPPPY motif at 702- 707aa sequence) predicted a possible interaction between Smurf2 and CNKSR2 [see Additional file 1: Table S1] which was further confirmed by docking studies between Smurf2-WW2/3 and CNKSR2 ‘SPPPPY’ motifs [see Additional file 2: Table S2]. As shown in Figure 11, WW2 domain of Smurf2 demonstrates a higher penetration and stabilization with ‘SPPPPY’ motif of CNSRK2 compared with Smurf2-WW3 domain. Moreover, we observed that depletion of Smurf2 caused a more rapid degradation of CNKSR2 during the cycloheximide chase assay (Figure 12D). This correlation of Smurf2 with CNKSR2 may explain the role of Smurf2 in proliferation and invasiveness of tumor cells.

Recent studies have shown that downregulation of CNK (Connector enhancer of KSR) proteins which are scaffold proteins regulating mitogen-activated protein kinase pathways, diminishes the proliferation and invasiveness of cancer cells, particularly breast cancer cells [21,35]. Remarkably, we also observed that Smurf2 knockdown caused a considerable downregulation in the expression of CNKSR2, a CNK homolog, followed by a concomitant decrease in the proliferation of breast cancer cells (Figures 7 and 8). To further elucidate the mechanism of this anti-proliferation effect, cell cycle analysis was conducted. An accumulation of Smurf2 knockdown cells were observed in the G_0_/G_1_ phase compared with control cells (Figure 9). All these data strongly suggest the role of Smurf2 in breast cancer proliferation.

G1–S progression is regulated by the controlled expression and activity of different cyclins (cyclin D, E and A), Cdks (Cdk 4, 6 and 2), CKIs (INK4 and cip/kip family of proteins), Rb protein and E2F transcription factor [36]. Moreover, we observed that expression of cyclin D1, a critical regulator of G1 phase progression of breast cancer cells was found to be significantly downregulated in Smurf2 siRNA treated cells (Figure 10). There is mounting evidence that cyclin D1 plays a critical role in breast cancer cell cycle control. The induction of cyclin D1 in breast cancer cells shortens the G1 phase and increases the number of cells that progress through the G1 phase, resulting in an increased proliferation [20]. In mammalian cells, the cyclins associate with specific cyclin-dependent kinases such as Cdk2, Cdk4, and Cdk6 which are key regulators of G1 to S phase transition [37]. In our study we observed that expression of Cdk4, one of the consistent partners of cyclin D1 was found to be inhibited in cells treated with Smurf2 siRNA, followed by a concurrent downregulation in the expression of Cdk1, pCdk1 and Cdk2. Besides cyclins and Cdks, the cyclin-dependent kinase inhibitors (CKIs), specifically, the Cip/Kip family CKIs such as p21 ^WAF1/CIP1^ and p27 ^KIP1^ regulates G1-S phase progression by inhibiting the cyclinD-cdk4/cdk6 association [37]. Consistently, our studies have shown that Smurf2 knockdown caused a marked upregulation in the expression of p21 and p27 thereby promoting accumulation of cells in the G1 phase (Figure 10).

The PI3K/AKT-signaling pathway regulates proliferation as well as cell survival and is constitutively activated in various human cancers, including breast cancer. An accumulation of evidence supports a key role for the PI3K pathway in cell cycle progression especially during the G1-S transition. A key effector of this pathway is AKT [5,21]. Fritz et al. reported that CNKSR2, the human homolog most resembling Drosophila CNK, modulates the Raf–MEK–ERK and NF-kB pathways in neuronal cells. The role of hCNKSR2 in RTK-mediated events has been examined in PC12 cells. Interestingly, knockdown of hCNK2 in PC12 prevented NGF-dependent ERK activation as well as neurite outgrowth. It thus appears that CNK proteins can mediate RTK-specific signals and that their function is not restricted to RAS/ERK signaling [35]. In our study we observed that downregulation of CNKSR2 expression following Smurf2 knockdown did not affect the MEK, ERK and NF-kB signaling cascades. However, there is a marked reduction in the expression of pAKT(S473) and total AKT levels. In addition, the PI3K catalytic subunit p110 was found to be moderately downregulated in Smurf2 knockdown cells, however there is not much variation in the expression levels of the regulatory subunit p85 which is required for the stabilization and localization of p110-PI3K activity (Figure 13).

Liang et al. reported that proteolysis of c-Myc and cyclin D1, which play distinct roles in cell cycle progression through G1 phase is regulated by the PI3K-AKT pathway. The stability of c-Myc is controlled by phosphorylation at S62 and T58 in a hierarchical fashion and AKT activation would stabilize c-Myc through inhibition of GSK-3β mediated T58 phosphorylation thereby protecting it from ubiquitin dependent degradation. In addition overexpression of constitutively active AKT was shown to extend the half-life of cyclin D1 protein whereas PI3K inhibition accelerated cyclin D1 degradation [25]. Consistently, we also observed that Smurf2 mediated CNKSR2 dependent downregulation of AKT activation caused inhibition of activation of c-Myc and cyclin D1 (Figure 13) thereby promoting accumulation of Smurf2 knockdown cells in the G0/G1 phase. Recently, Guo et al. reported that c-Myc can indirectly inhibit PTEN expression in GBM cells, thereby promoting proliferation. MMAC/PTEN phosphatase is a critical mediator of PI3K-AKT signaling that dephosphorylates the phosphoinositide products of PI3-kinase and functions as its natural antagonist [31,32]. In our study we observed that downregulation of c-Myc was followed by a concomitant increase in the expression of PTEN (Figure 13) which might be responsible for the decreased phosphorylation of AKT at S473 which is consistent with the well-established inverse relationship between MMAC/PTEN expression and AKT activation.

PI3K-AKT promotes cell survival by indirectly regulating the phosphorylation of various downstream signaling and target molecules including the mammalian forkhead box subgroup ‘O’ (FoxO) of forkhead transcription factors consists of FoxO1, FoxO3a, FoxO4 and FoxO6 which play an important role as tumor suppressor in several human malignancies. Specifically, FoxO3a activity is negatively regulated by AKT, which phosphorylates FoxO3a at multiple sites, facilitating its association with 14-3-3 protein, thereby leading to its transport out of the nucleus and retention in the cytoplasm. The cytosolic retention of FoxO3a prevents the transactivation of downstream target genes such as p27/Kip1 [30]. We hypothesized that inhibition of AKT phosphorylation mediated by downregulation of expression of CNKSR2 in Smurf2 knockdown cells would lead to nuclear sequestration of FoxO3a and increased transcription of responsive genes. Studies have shown that FoxO3a is dephosphorylated and activated by LY294002, which correlates with upregulation of p27/Kip1 [30]. In agreement with the hypothesis, our results demonstrate that Smurf2 knock down caused an upregulation in the expression of FoxO3a. However, the expression of phosphorylated FoxO3a(S253) was decreased after Smurf2 knock down resulting in the nuclear retention of these proteins and increased transcription of responsive genes such as p27/Kip1 (Figure 13).

Together, these data suggest that Smurf2 knockdown modulates the proliferation and invasiveness of breast cancer cells via regulating the PI3K-AKT signaling pathway and its downstream targets in a CNKSR2 dependent manner. Increased levels of CNK homologs have been identified in various cancers including breast cancer [27]. CNK1 was identified as one of a few critical genes that mediate metastasis in breast cancer [35]. However, the functional significance and regulation of CNKSR2 which is specifically involved in neuronal differentiation has not been fully identified yet. In our study, we report for the first time that Smurf2 knockdown caused a marked decrease in the expression of CNKSR2 which in turn downregulates the proliferation and invasiveness properties of breast cancer cells via the PI3K-AKT signaling cascade. It will be important to determine whether Smurf2 can interact with CNKSR2 which possess a PPxY sequence in its structure which is necessary for interaction with WW domain of Smurf2 [34] and whether its levels correlate with each other in human breast cancer progression models. Future studies using human cancer specimens should provide insight into the putative oncogenic interaction of these two proteins in the regulation of cell cycle progression and cell proliferation of breast cancer cells.

## Conclusions

In summary, studies from our laboratory have shown that silencing of Smurf2 with siRNA resulted in significant inhibition of focus formation potential, anchorage-independent growth capability, migration, invasiveness, and proliferation in breast cancer cells by a possible interaction with CNKSR2. The expression of CNKSR2, a multi-functional scaffold protein involved in Ras-Raf signaling cascade was inhibited following Smurf2 knockdown which modulates the PI3K-AKT signaling pathway and its downstream molecular targets involved in cell proliferation and invasiveness. We therefore conclude that targeting the Smurf2-CNKSR2- PI3K/AKT functional axis could be used as a potential preventive/therapeutic strategy in the management of breast cancer in humans.

## Methods

### Cell lines and culture conditions

All human breast cancer cell lines (MCF 10A, MCF-7, MDA-MB-231, MDA-MB-435, T-47D, NCI/ADR-RES, SK-BR3 and Hs578T) were purchased from the ATCC (Rockville, MD). MCF10A cells were cultured in DMEM/F12 supplemented 10% FBS, 20 ng/ml EGF, 100 ng/ml cholera toxin, 0.01 mg/ml insulin, and 500 ng/ml hydrocortisone and all other cell lines were maintained in DMEM medium supplemented with 10% FBS, penicillin (100unit/ml), and streptomycin (100 μg/ml) and were cultured at 37°C in a humidified atmosphere containing 5% carbon dioxide. The culture medium was changed every other day and the cells were passaged when they reached 80 to 90% confluency.

### siRNA -directed gene knockdown

Cells were transfected with siRNA against Smurf2, in the form of a mixture of three target specific 20–25 nt siRNAs targeting different regions of Smurf2 (41675, Santa Cruz Biotechnology) or the negative control siRNA (37007, Santa Cruz Biotechnology) included in the kit and siLentFect (170–3361, Bio-Rad) according to the manufacturer’s instructions. Briefly, cells were seeded in a 6 well plate at a density of 2 × 10^5^ cells/well in 2 ml antibiotics-free normal growth medium supplemented with FBS medium 24 h before the transfection. Eight microliters (80 pmols) of the siRNA were mixed with 2 μl siLentFect in 200 μl Opti-MEM (Gibco/Invitrogen) medium and were incubated at room temperature for 45 min to form a complex. After washing cells with Opti-MEM, the 200 μl transfection mixtures were added to each well with 800 μl Opti-MEM medium. Twenty-four hours after the transfection, the medium was replaced with fresh DMEM medium containing 10% FBS. Cells were collected at 36 or 48 h for RNA or protein isolation.

### Reverse transcriptase -PCR

Total RNA was isolated using TRIzol (Invitrogen) reagent according to the manufacturer’s protocol. 10 μg of total RNA was converted to cDNA using oligo-dT primer and M-MLV Reverse Transcriptase (Promega) in a 25 μl reaction. The RT-PCR reaction mixture contained 5 μl of 10× reaction buffer, 2 μl of cDNA template, 1 μl each of forward and reverse primers, and 0.5 μl of Taq DNA polymerase (Sigma) in a final volume of 20 μl. The reaction was done at 94°C for 5 min (Initial denaturation), 94°C for 45 s (Denaturation), 60°C for 1 min (Annealing), 72°C for 45 s (Extension), and 72°C for 5 min (Final extension) for 35 cycles. Analysis of amplified products was done on 2% agarose gel and visualized using Fluor-S™ MultiImager (Bio-Rad). The PCR products were quantified by densitometric analysis, using Bio-Rad Quantity One software. A 100-bp ladder (New England Biolabs) was used as a size standard. The primers used for the study included: Smurf2, 5′-CGCTTGATCCAAAGTGGAAT-3′ (forward) and 5′-GGTTGATGGCATTGGAAAGA-3′ (reverse); CNKSR2, 5′-TGGTCCCCACTGATCTTCTC-3′(forward) and 5′-TGAGCAAATGGTCTCCGAGT-3′(reverse). GAPDH was used as an internal control and the primers used were 5′-TTAAAAGCAGCCCTGGTGAC-3′ (forward) and 5′-CTCTGCTCCTCCTGTTCGAC-3′ (reverse).

### Quantitative RT-PCR

Quantitative real-time RT-PCR was performed as described previously [9]. Total RNA was isolated from cells using TRIzol reagent (Invitrogen) according to the manufacturer’s protocol. Total RNA (2 μg) was converted to cDNA using oligo dT primer and M-MLV Reverse Transcriptase (Promega). One hundred nanograms of cDNA was amplified and detected with the Power SYBR Green PCR Master Mix (Applied Biosystems) on an ABI 7900HT Fast Real-Time PCR System (Applied Biosystems). To confirm the PCR specificity, PCR products were subjected to a melting-curve analysis. The comparative threshold method was used to calculate the relative amount of mRNA of treated sample in comparison with control samples after normalization with the GAPDH control. The primers used for the study included: Smurf2, 5′-TGGATCAGGAAGTCGGAAAA-3′(forward) and 5′- GGACATGTCTAACCCCGGA-3′(reverse); CNKR2, 5′- AACCGGTGAGCAAATGGTCT-3′(forward) and 5′-CCTGATGTGTAATGCGCAGC-3′ (reverse). We used GAPDH as an internal control and the primers used were 5′-ATGGGGAAGGTGAAGGTCG-3′ (forward) and 5′-GGGGTCATTGATGGCAACAATA-3′(reverse).

### Western blot analysis

The cultured cells were washed twice with ice-cold PBS and lysed on ice in lysis buffer comprising 10% NP40, 5 M NaCl, 1 M HEPES, 0.1 M DTT, 0.1 M EGTA, 0.1 M EDTA, protease inhibitors (Sigma) and differential centrifugation (14000 rpm for 10 minutes). The amount of total protein was determined using Bradford’s assay (Bio-Rad). An equal amount of total protein (60 μg) was loaded and separated by SDS-PAGE. The separated proteins were electrotransferred onto nitrocellulose membrane (Amersham Pharmacia Biotech), blocked with 5% skimmed milk and probed with appropriate antibodies. The protein was then visualized using horseradish peroxidase-conjugated secondary antibodies (Santa Cruz Biotechnology) and immunoreactive bands were developed with an ECL system (Amersham Pharmacia Biotech) and quantified using Image Lab Software version 4.1(BIO RAD). The antibodies used were Smurf2(H-50), cyclin D1(A-12), cyclin A(BF683), cyclin E(C-19), cyclin B1, cdk4(B-10), p21(187), p27(C-19), MEK1/2(12B), ERK1/2, NF-kBp65(C-20), IkB-α(H-4), c-Myc(9E10), PTEN(B-1), PI3Kp110, Goat anti-rabbit IgG, and Goat anti-mouse IgG from Santa Cruz Biotechnology (Santa Cruz, California); Cdk2, pMEK1/2(S217/221), pERK1/2(Thr202/Tyr204), Akt, pAkt(S473), 14-3-3, FoxO3a(75D8), pFoxO3a(S253) from Cell Signaling Technology (Beverly, Massachusetts); CNKR2 from Abcam (Cambridge, UK); PI3Kp85 from BD Biosciences (New Jersey, USA); and b-Actin (Clone AC-15) from Sigma Aldrich (St. Louis, USA).

### Cycloheximide chase assay

A cycloheximide chase assay was performed as described [9]. Briefly, MDA-MB-231 cells were transfected with Smurf2 siRNA or nontargeting siRNA 48 h before the experiment. Cycloheximide (Sigma) was added to the cells at 100 μg/ml final concentration. Cells were harvested 0, 1, 2, or 3 h after cycloheximide addition and the cell lysate was subjected to SDS-PAGE followed by immunoblotting with specific antibodies.

### MTT assay

Cell proliferation was examined by MTT assay. Briefly, MDA-MB-231 cells were seeded at a density of 5 × 10^3^ cells/well in 96-well plates in triplicate, each contained 100 μl of medium.

At different times (24, 48, and 72 h), after Smurf2 siRNA transfection, cells were incubated with 10 μl of 5 mg/ml 3-(4,5-dimethylthiazol-2-yl)-2,5-diphenyltetrazolium bromide (MTT; Sigma) diluted in PBS at 37°C. Four hours later, 200 μl of isopropanol was added to the MTT treated wells and the absorption at 570 nm was determined using a benchmark microplate reader (Bio-Rad).

### BrdU incorporation assay

A BrdU cell proliferation assay was performed according to the manufacturer’s instructions. MDA-MB-231 cells (2 × 10^4^ cells/well) were incubated in 96-well plates each contained 100 μl of medium. At different times (24, 48, and 72 h), after Smurf2 siRNA transfection, cells were incubated with 30 μM 5-bromo-2′-deoxyuridine (BrdU; Sigma) for 30 min at 37°C. Cells were fixed and permeabilized with methanol, treated with HCl, neutralized, and blocked with 2% BSA in PBS. Cells were then incubated with mouse anti-BrdU (70443, Santa cruz Biotechnology), FITC-conjugated anti-mouse (2010, Santa cruz Biotechnology). The absorbances of the wells were read at 520 nm on an automatic microplate reader (TECAN infinite 200).

### Clonogenic assay

Smurf2 knockdown MDA-MB-231 cells and control transfected cells (1x10^3^ cells/well) were seeded in six-well plates in DMEM/5% FBS. The medium was changed every 2 days. Cells cultured for 14 days were washed twice with 1xPBS, fixed by 4% paraformaldehyde, and stained with 0.5% crystal violet and colonies containing more than 50 cells (established by microscopy) were counted manually. Images of the colonies were obtained using a digital camera. The experiments were done in duplicate at least three times.

### Soft agar assay

Colony formation ability was examined by anchorage independent soft agar assay on MDA-MB-231 and MCF7 cells. Briefly, 1.5 ml FBS supplemented medium containing 0.8% agarose were added in 35-mm cell culture dishes and allowed to solidify (base agar). Next, 1×10^3^ Smurf2 knockdown MDA-MB-231 and MCF7 cells were mixed with 1.5 ml FBS supplemented medium containing 0.35% agarose and added to the top of base agar. The cells were then cultured for 21 days at 37°C under 5% carbon dioxide. The dishes were stained with 0.01% crystal violet, and the colonies were examined with microscope. The experiments were done in triplicate at least two times.

### Wound healing assay

MDA-MB-231cells seeded in 12-well plates (2 × 10^5^ cells/well) were transfected with Smurf2 siRNA as described above. Once the cells reached 90% confluency, a wound area was carefully created by scraping the cell monolayer with a sterile 100 μl pipette tip. After being washed three times with PBS, scratches including the flanking front lines of cells, were photographed (20-fold magnification). Subsequently, the cells were incubated at 37°C in 5% CO_2_. The width of the wound area was monitored with an inverted microscope at various time points. Wound closing was compared between Smurf2 knockdown cells and control transfected cells after measuring the wound width and evaluated using Leica application Suite software (LASV3.8, Germany). Differences between the data points were determined by Student’s *t* test where p < 0.05 was considered significant. Experiments were performed independently two times, evaluating 4 – 8 scratches in each experiment.

### Migration and invasion assay

Cell migration and invasion assay was done as described previously [14]. Briefly, MDA-MB-231 cells were seeded at 2×10^5^ cells/well in 6-well format and transfected by Smurf2 siRNA as above. Forty-eight hours after the transfection, the cells were trypsinized and resuspended in FBS-free DMEM medium. For the migration assay, a total of 1 × 10^5^ cells were added to the top chamber of pre-wet transwell inserts (BD Falcon™ 8 μm Control insert). For the invasion assay, 1 × 10^5^ cells were plated in the top chamber of matrigel-coated transwell inserts (BD Falcon™ 8 μm Control insert). Complete culture media with 10% FBS was added to the lower chamber as a chemoattractant. After incubation for 16 h (migration assay) or 24 h (invasion assay), cells were washed, fixed with 10% formaldehyde for 20 min, and stained with 0.5% crystal violet. Cells that did not migrate through the pores were mechanically removed by a cotton swab. The images of migrated cells were acquired by an inverted microscope with a magnification of 200×. The number of migrated or invaded cells was counted from five or six randomly selected fields in a blind way. All migration experiments were performed in triplicates and repeated three times.

### Cell cycle analysis

Cell cycle distribution after Smurf2 knockdown was carried out at indicated times. Cells were harvested and fixed with ice-cold 70% ethanol and precipitated overnight at 4°C. After fixation, cells were resuspended in room temperature PBS, treated with RNase A (100 μg/ml), and incubated for 1 h at 37°C followed by treatment with propidium iodide (10 μg/mL), in the dark, for 15 min. Finally, DNA content of the cells was analyzed using FACS Aria (Special order system, BD, USA).

### Immunofluorescence

Cells at 60% confluence were plated onto sterilized glass coverslips. The slides were washed in phosphate-buffered saline (PBS) and fixed for 20 min in ice-cold acetone/methanol (1:1) on ice. The slides were then blocked with 3% BSA in PBS for 1 hour at 37°C, followed by incubation with anti-Smurf2 (25511, Santa cruz Biotechnology), anti-Ki67 (H300, Santa cruz Biotechnology), FITC-conjugated anti-rabbit, and PE-conjugated anti-mouse (2012, Santa cruz Biotechnology) antibodies. Cells were also stained with Hoechst dye (Hoechst 33342, Invitrogen) to reveal nuclei. The images were taken at × 60 magnification using a confocal microscope (FV1000 Olympus).

### Homology based approach to predict novel interacting partner of Smurf2

There are many different computational approaches to predict protein interactions; some are based on genomic context, co-evolution, co-expression or co-occurrence patterns of potentially interacting partners. In order to predict novel direct interaction partners for Smurf2, we used a simple homology based approach [38]. We first used four known interactors of Smurf2 (Smad2, Smad6, Smad7, and NDFIP1) and extracted the sequences and carried out a secondary structure prediction. We then wrote a Perl script that searched for sequences in Uniprot that most closely matched the known Smurf2 binding motif containing the PY motif and had the same secondary structure neighborhoods as the identified ones. BLASTp with p-value cut-off of 0.01 was used for sequence based searches and 3D-PSSM was used for secondary structure assignment. Details of the test cases that were used to arrive at the secondary structure consensus and the predicted protein interaction partners are given (see Additional file 1: Table S1). The locations of the PY motif, known to be involved in WW domain binding are also shown. We included only those proteins that matched both criteria of a secondary structural match (beta-strand flanking region or having no secondary structure) and a strong PY motif match.

The ‘SPPPPY’ motif of CNKSR2 was modeled by submitting the sequence to I-TASSER prediction server and Patchdock, which detects shape complimentary of molecular surfaces was used determine the interaction between CNKSR2 and Smurf2 WW2/3 domains. The scoring is based on the distance of ligand atoms from the protein surface, close atoms receive a positive score, and penetrating atoms receive a negative score, respective to penetration depth. The docking was performed focusing a specific area of the CNKSR2 (‘SPPPPY’ motif) by mentioning the residues in the parameter file. Out of all the solutions generated by Patchdock, the one having the highest score proceeded to the simulation studies. OPLS-AA(2001) forcefield in Gromacs 4.5.4 was used for simulating the CNKSR2- Smurf2WW2/3 complexes. The complexes were placed in a water box. All runs were at 300 K with a time step of 2 fs. All bonds were constrained using the LINCS algorithm. The simulation was performed for 500 ps. The energy analysis was done using the tools available in GROMACS package.

### Statistical analysis

Data were expressed as the mean ± standard deviation. Difference between any two groups was determined by analysis of variance. P < 0.05 was considered statistically significant.

## Abbreviations

Smurf2: Smad ubiquitin regulatory factor 2; HECT: Homologous to the E6-AP carboxyl terminus; TGF- β: Transforming growth factor β; BMP: Bone morphogenetic pathway; siRNA: short interfering RNA; Nedd4: Neural precursor cell expressed developmentally down-regulated protein 4; Mad2: Mitotic arrest deficient 2; NEDD9: Neural precursor cell expressed developmentally down-regulated protein 9; RhoA: Ras homology gene family member A; KLF2: Kruppel-like Factor 2; KLF5: Kruppel-like Factor 5; PCNA: Proliferating cell nuclear antigen; Ki67: Kiel-67 antigen; CDKs: Cyclin dependent kinases; Ras and Raf: small GTPase; MEK: Mitogen-activated protein kinase kinase; ERK: Extracellular signal-regulated kinases; NF-kB: Nuclear factor kappa-light-chain-enhancer of activated B cells; PI3K: Phosphoinositide 3-kinase; Akt: Protein kinase B; CNKSR2: Connector enhancer of kinase suppressor of Ras 2; RTK: Receptor tyrosine kinases; FoxO3a: Forkhead box O family of transcription factors; c-Myc: Proto-oncogene; PTEN: Phosphatase and tensin homolog; GSK-3β: Glycogen synthase kinase-3 beta; MTT: [3-(4,5-dimethylthiazol-2-yl)-2,5-diphenyl tetrazolium bromide; BrdU: 5-bromo-2′-deoxyuridine; NDFIP1: Nedd4 family-interacting protein 1.

## Competing interests

The authors declare that they have no competing interests.

## Authors’ contributions

AN and DD designed the research. DD performed the research and wrote the paper. DD and SJ analyzed the data. RH carried out the *in silico* studies. All authors read and approved the final manuscript.

## Supplementary Material

Additional file 1: Table S1 Homology based prediction of Smurf2 and CNKSR2 interaction. CNKSR2 possess a ‘SPPPPY’ motif at 702–707 sequence region that shows a strong PY motif match with the WW domain of Smurf2 compared with other known interacting partners such as Smads (Smad2, 6 and 7) and NDFIP1(Nedd4 family interacting protein 1). Click here for file

Additional file 2: Table S2 Smurf2 WW2/3 and CNKSR2 ‘SPPPPY’ domain docking. Docking of Smurf2 WW2/3 and CNKSR2 ‘SPPPPY’ domains using PATHDOCK and GROMACS indicates that CNKSR2-Smurf2WW2 docking shows highest score, area of interaction and more penetration and stabilization (less energy) with ‘SPPPPY’ motif (702–707 sequence) of CNSRK2 compared with CNKSR2-Smurf2WW3 docking. Click here for file
