# Supplementary material for: Smurf2 E3 ubiquitin ligase modulates proliferation and invasiveness of breast cancer cells in a CNKSR2 dependent manner
Source: Cell Div. 2014 Aug 31;9:2. doi: 10.1186/1747-1028-9-2 (PMC4154384; doi:10.1186/1747-1028-9-2)
Supplement: Additional file 1: Table S1 — Homology based prediction of Smurf2 and CNKSR2 interaction. CNKSR2 possess a ‘SPPPPY’ motif at 702–707 sequence region that shows a strong PY motif match with the WW domain of Smurf2 compared with other known interacting partners such as Smads (Smad2, 6 and 7) and NDFIP1(Nedd4 family interacting protein 1). [file 1747-1028-9-2-S1.docx]

| **Sl. No** | **Proteins, status of interaction with Smurf2** | **Secondary structure (SS) flanking the PY motif** | **Sequence and location of the PY motif** |
| --- | --- | --- | --- |
| 1 | Smad6 (known) | Beta strand loop beta strand | SPPPPY, 274- 279 |
| 2 | Smad7 (known) | No stable SS | SPPPPY, 206- 211 |
| 3 | Smad2 (known) | No stable SS | TPPPGY, 220- 225 |
| 4 | NDFIP1 (known) | No stable SS | APPPYS, 38- 43 |
| **5** | **CNKSR2 (predicted)** | No stable SS | **SPPPPY, 702- 707** |

**Table 1. Homology based prediction of Smurf2 and CNKSR2 interaction**
