# Supplementary material for: Smurf2 E3 ubiquitin ligase modulates proliferation and invasiveness of breast cancer cells in a CNKSR2 dependent manner
Source: Cell Div. 2014 Aug 31;9:2. doi: 10.1186/1747-1028-9-2 (PMC4154384; doi:10.1186/1747-1028-9-2)
Supplement: Additional file 2: Table S2 — Smurf2 WW2/3 and CNKSR2 ‘SPPPPY’ domain docking. Docking of Smurf2 WW2/3 and CNKSR2 ‘SPPPPY’ domains using PATHDOCK and GROMACS indicates that CNKSR2-Smurf2WW2 docking shows highest score, area of interaction and more penetration and stabilization (less energy) with ‘SPPPPY’ motif (702–707 sequence) of CNSRK2 compared with CNKSR2-Smurf2WW3 docking. [file 1747-1028-9-2-S2.docx]

**Table 2. Smurf2 WW2/3 and CNKSR2 ‘SPPPPY’ domain docking**

| **Interacting motifs** | **Score** | **Penetration** | **Area of interaction** | **Total energy kJ/mol** |
| --- | --- | --- | --- | --- |
| CNKSR2-WW2 | 10306 | -3.04 | 1570.10 | -198501.6 |
| CNKSR2-WW3 | 7982 | -3.06 | 1149.80 | -196649.3 |
